# On the stability of stalled RNA polymerase and its removal by RapA

**DOI:** 10.1093/nar/gkac558

**Published:** 2022-07-12

**Authors:** James R Portman, M Zuhaib Qayyum, Katsuhiko S Murakami, Terence R Strick

**Affiliations:** Institut de Biologie de l’Ecole Normale Supérieure, PSL Université, INSERM, CNRS, Paris 75005, France; Horizons 2020 Innovative Training Network, DNAREPAIRMAN, Paris 75005, France; Department of Biochemistry and Molecular Biology, The Center for RNA Molecular Biology, The Center for Structural Biology, Pennsylvania State University, University Park, Pennsylvania 16802, USA; Department of Biochemistry and Molecular Biology, The Center for RNA Molecular Biology, The Center for Structural Biology, Pennsylvania State University, University Park, Pennsylvania 16802, USA; Institut de Biologie de l’Ecole Normale Supérieure, PSL Université, INSERM, CNRS, Paris 75005, France; Horizons 2020 Innovative Training Network, DNAREPAIRMAN, Paris 75005, France; Equipe Labellisée de la Ligue Nationale Contre le Cancer, Paris 75013, France

## Abstract

Stalling of the transcription elongation complex formed by DNA, RNA polymerase (RNAP) and RNA presents a serious obstacle to concurrent processes due to the extremely high stability of the DNA-bound polymerase. RapA, known to remove RNAP from DNA in an ATP-dependent fashion, was identified over 50 years ago as an abundant binding partner of RNAP; however, its mechanism of action remains unknown. Here, we use single-molecule magnetic trapping assays to characterize RapA activity and begin to specify its mechanism of action. We first show that stalled RNAP resides on DNA for times on the order of 10^6^ seconds and that increasing positive torque on the DNA reduces this lifetime. Using stalled RNAP as a substrate we show that the RapA protein stimulates dissociation of stalled RNAP from positively supercoiled DNA but not negatively supercoiled DNA. We observe that RapA-dependent RNAP dissociation is torque-sensitive, is inhibited by GreB and depends on RNA length. We propose that stalled RNAP is dislodged from DNA by RapA via backtracking in a supercoiling- and torque-dependent manner, suggesting that RapA’s activity on transcribing RNAP *in vivo* is responsible for resolving conflicts between converging polymerase molecular motors.

## INTRODUCTION

RNA polymerase is characterized by a very stable association to DNA in the elongation phase, made possible in part by the closure of the RNAP’s ‘crab-claw’ or ‘clamp’ domains about the DNA (1). Indeed, for RNAP to reliably transcribe full-length mRNA its average processivity must be much greater than the longest genes. As a result, RNAP stalled during elongation does not easily dissociate from DNA but instead forms possibly the most stable, formidable obstacle to other proteins engaged with the DNA such as DNA polymerases, repair proteins and recombinases. Because of this stability, the dissociation of a stalled RNAP from DNA absent accessory factors has not been quantitatively characterized. The presence of numerous factors whose main purpose is to remove or remodel stalled RNAP shows us firstly that these complexes are indeed very stable, and secondly that there is a pressing need to remove them from DNA in a timely manner, to repair the stall cause and/or to enable other motors access to DNA (2–4).

RapA was the first bacterial homolog found to be of the SWI/SNF family of eukaryotic proteins that are generally associated with chromatin remodeling (5). The RapA protein was identified because it consistently co-purifies with RNAP preparations from *Escherichia coli* (5–7). RapA is a seven-domain 110-kDa protein with RNAP-binding, nucleic acid-binding and ATPase activities (8–12). RapA binds to core RNAP near the RNA exit channel and this binding stimulates RapA ATPase activity (8,11). It has been shown that RapA activates transcription *in vitro*, but RapA does not affect promoter binding, promoter escape, elongation nor termination (11). It has been speculated that RapA activates transcription by recycling RNAP that is in an off-pathway state (11). Further studies showed the existence of a transient RapA–RNA interaction, leading to the hypothesis that RapA may remodel the RNA in the off-pathway post-termination complex which ultimately leads to complex dissociation (9,13). Little is understood on the precise mechanism of RapA action, and furthermore the presence and biological importance of such atypical RNAP states *in vivo* is not known.

In this study we use a single-molecule approach to interrogate the mechanism by which stalled RNAP is removed from DNA. We show that RNAP stalled on positively supercoiled DNA is removed when very high torque is applied, and that RapA accelerates this process. It appears that the torque-dependence of the kinetics of RNAP removal are similar when RapA is absent or present, indicating that there is a common underlying mechanism. This removal occurs only on DNA which is positively supercoiled and is never detected on DNA that is negatively supercoiled. We propose RapA removes RNAP by backtracking because first RapA is functionally inhibited by the anti-backtracking factor GreB, second it takes RapA progressively longer to remove RNAP stalled progressively further downstream from the promoter, third the addition of RNAse A ablates this length-dependence, and fourth we can directly observe backtracking of bead-labeled RNAP along DNA upon addition of RapA. Our data leads us to the conclusion that the biological role of RapA has evaded detection primarily because it acts on RNAP, on meaningful timescales, in specific transient topological environments.

## MATERIALS AND METHODS

### DNA constructs

The DNA construct allowing us to stall RNAP +20 base-pairs from the transcription start site (TSS) by transcribing with only ATP, UTP, and GTP is part of a home-made vector, described in (3) (referred to here as pET21ΔMCS_RPOC_20stall). Briefly, the RPOC gene from *Thermus aquaticus* was inserted into the pET21ΔMCS vector. Then, a transcription cassette was inserted in the unique KpnI site of the RPOC gene. The sequence of the transcription cassette is as follows (promoter elements underlined and stalling site in bold):

5′GGTACCTCGAGGGAATCATAAAAAATTTATTTGCTTTCAGGAAAATTTTTCTGTATAATAAGCTTATAAATTTGAGAGAGGAGA**CC**AAATATGGCTGGTTCTCGCACTAGTTCCGAATAGCCATCCCAATCGAACAGGCCTGCTGGTAATCGCAGGCCTTTTTATTTGTGACCCCGGGTAGAATTCGGTACC3′

A PCR is performed on this vector using PCR primers bearing one Xba and one Sbf restriction site, enabling subsequent ligation of the 2kb PCR product into a pUC18 vector. This vector was then digested with XbaI and SbfI, and the 2kb band gel purified.

This DNA was ligated at the XbaI end to a 1kb DNA handle bearing many biotin groups on both strands, and ligated at the SbfI end to a 1kb DNA handle bearing many digoxigenin groups on both strands. These handles are home-made, using the *Thermus aquaticus* RPOC gene as template for a PCR reaction using the following primers:

5′GAGAGACCTGCAGGACATCAAGGACGAGGTGTGGG3′

5′GAGAGATCTAGATCCTCAAAGTTCTTGAAGACCGCCTGG3′

The PCR reaction is done twice, one with dUTP-biotin in the dNTP mix, and the other with dUTP-digoxigenin in the dNTP mix. The biotin-labeled DNA was cut with XbaI whilst the digoxigenin-labeled DNA was cut with SbfI-HF.

Constructs stalling the RNAP +36 base-pairs from the TSS and +83 base-pairs from the TSS were generated by altering the parent pET21ΔMCS_RPOC_20stall construct. In both cases, this vector was digested with HindIII and SpeI, and the open vector was gel-purified. The following DNA fragments were ligated into the open vector to generate the relevant construct:

36_stall:

5′AGCTTATAAATTTGAGGAGAATAATTGTAGAGGAGAGAGTCCAAATATGGCTGGTTCTCGCA3′

83_stall:

5′AGCTAATAAATTTGAGGAGACCAAATATGGCTGGTTCTCGACGGTCTTCTCCATGCCCAGGCGAAGCTTAGAGAAA3′

The 36_stall construct shifts the stall site from +20 to +36. The 83_stall construct was designed to destroy the original HindIII site and create a new one further downstream, enabling the insertion of the CPD-bearing oligo by restriction-digestion and ligation using the HindIII and SpeI sites. The sequence of the CPD-bearing oligo is:

5′CTAGAGGAGAACCAGCCATATTTXXTCTCCTCTCTCAAATTTATT3′ (where XX is the CPD)

Therefore, the 83_stall construct allows us to stall RNAP at +15, wash out all remaining RNAP, before adding ATP, UTP, GTP and CTP to enable transcription up to the stalling CPD at +83.

The 8kb DNA construct used for tethered-RNAP experiments was generated as previously described (3,14). It is ligated at one end to a 1 kb digoxigenin-labeled DNA fragment. The promoter is roughly 1 kb from the opposite end and there is no terminator.

Ligated DNA constructs are stored at −20°C, with a working stock of 50pM stored at 4°C. This DNA is ligated rapidly first to streptavidin-coated magnetic beads, and then added to the glass coverslip. The DNA-bead mixture incubates on the surface for 10 min, after which free magnetic beads and free DNA are washed out with buffer.

### Hybridising oligonucleotide

The ssDNA oligonucleotide that hybridises to the nascent RNA of RNAP stalled at +36 had the following sequence:

5′ATTCTCCTCAAATTTAT3′

In experiments using this oligonucleotide, first RNAP, ATP, UTP, GTP, and the oligonucleotide were added. RNAP was observed to stall at +36, and then free RNAP, free oligonucleotide and free NTPs were washed out before RapA and ATP were added.

### Proteins

*E. coli* core RNAP, biotinylated-RNAP, GreB and σ^70^ were purified as previously described and core RNAP was saturated with a fivefold excess of σ^70^ (3,15). *E. coli* RapA was purified from BL21(DE3) cells transformed with pQE80L expression vector (encoding N-terminally His_6_-tagged full-length RapA) grown at 37°C to OD = 0.6 and induced using 1mM IPTG for 4 h at 30°C (16,17). Cells were lysed at 4°C in lysis buffer (10 mM Tris pH 8.0 (at 4°C), 5% glycerol, 1M NaCl, 0.5mM beta-mercaptoethanol, and Complete protease inhibitor) using an Avestin C5 homogeniser and samples were kept at 4°C for the duration of the purification. Clarified cell lysate was applied to a HisTrap IMAC column, washed with up to 15 mM imidazole and eluted with 200 mM imidazole. The eluate was applied to a HiTrap Q HP column, washed and eluted over a 0–500 mM NaCl gradient with RapA eluting at ∼300 mM NaCl. The eluate was concentrated and run on a gel-filtration column (Superdex200 16/60) equilibrated in 10 mM Tris pH8.0 (at 4°C), 5% glycerol, 100 mM NaCl, 0.1 mM EDTA and 5 mM 1,4-dithiothreitol. Single-use 10 μl aliquots were snap-frozen in liquid nitrogen and stored at −80°C.

### Flow cell preparation

Flow cells derivatized with anti-digoxigenin were prepared as previously described (18).

### Reaction conditions

Single-molecule assays were performed at 34°C in reaction buffer containing 40 mM K-Hepes pH 8.0, 100 mM KCl, 8 mM MgCl_2_, 0.5 mg/ml BSA, 0.1% w/v Tween 20 and 1mM 1,4-dithiothreitol. Unless stated otherwise, concentrations of components in reactions were as follows: 100 pM RNAP holoenzyme, 100 nM RapA, 1 mM ATP, 200 μM GTP and 200 μM UTP. Collecting the specific number of events, *n*, typically requires more than three experimental runs involving normally 20–50 DNA molecules simultaneously.

### Tethered-DNA assays

Only single molecules of intact double-stranded DNA, verified by their ability to supercoil as per the expected rotation-extension curve (19,20), were retained for experimentation. For addition of components, DNA was positively supercoiled and the force was increased to ∼2 pN to prevent DNA molecules or beads becoming stuck to the surface under the flow of components. Once the flow ended the force was reduced and supercoiling returned to its initial value. For the low force ‘recycling assay’, DNA was maintained at a constant extending force of 0.3 pN at positive or negative supercoiling. For the ‘force-cycling’ assay, positively supercoiled DNA was kept at 0.3 pN for 400 s to allow RNAP to bind and stall, and then the force increased to the relevant value for many thousands of seconds.

### Tethered-RNA polymerase assay

To assemble the reaction, the 8 kb DNA construct was incubated at room temperature for 15 min with biotinylated-RNAP, ATP, UTP and GTP. Stalled elongation complexes were then bound to streptavidin-coated magnetic beads, and then deposited on to the anti-digoxigenin treated glass surface. The DNA-RNAP-bead mixture was incubated on the surface for 10 min, after which free magnetic beads and free DNA were washed out with buffer.

### Estimation of torque acting on supercoiled DNA

DNA extended by a constant force, F, and supercoiled so as to form interwound plectonemes experiences a constant torque, Γ, which can estimated using: Γ $ = \sqrt {2\xi .{k_B}.T.F}$ where ξ is the persistence length and 2ξ the Kuhn length, *k*_B_ is the Boltzmann constant, and T the temperature in Kelvins (21).

### Data acquisition and analysis

Histograms were fit to a single exponential distribution. Number of events (*n*) and the values of the fits with standard errors are stated in the figure legends. All data analysis was done using the PicoTwist software suite.

## RESULTS

### High torque removes stalled RNA polymerase from positively supercoiled DNA

**Figure 1.**
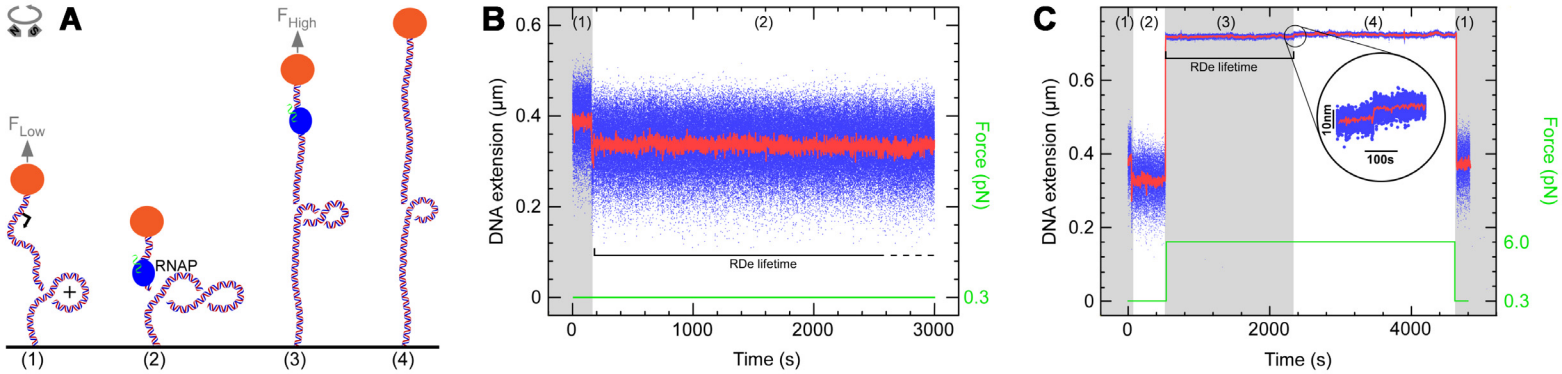
High torque removes stalled RNAP from DNA. (**A**) Experimental setup. The 2 kb DNA bears a promoter (arrow) and is tethered between a magnetically trapped bead (orange sphere) and glass surface (black line). DNA is positively supercoiled and at low force before components (RNAP and limiting NTPs) are added (state 1). RNAP binds to the promoter, initiates transcription, causing the DNA extension to decrease, and then stalls at +20. At low force it remains bound here indefinitely (state 2). The force is increased, causing the DNA molecule to extend and increase torque (state 3). RNAP dissociates from DNA, causing the DNA extension to increase (state 4). (**B**) Time-trace with RNAP and limiting NTPs on positively supercoiled DNA. As RNAP initiates transcription it scrunches and unwinds ∼2 turns of DNA, reducing DNA extension by ∼100 nm. Successful elongation proceeds with an unwound bubble of ∼9 base-pairs and so DNA extension is reduced by only ∼50 nm compared to baseline. As RNAP reaches +20 it stalls indefinitely. The number indicated at top refers to the states identified in (A). (**C**) Time-trace with RNAP and limiting NTPs in the ‘force-cycling’ assay. Positively supercoiled DNA is initially at low force (state 1). RNAP initiates transcription and stalls (state 2). The force is increased (state 3) and we observe RNAP dissociation (inset) as formation of state 4. RNAP dissociation is confirmed because when the system returns to the low force state, the DNA extension is the same as it was prior to RNAP binding (state 1).

To first establish the stability of stalled RNAP on DNA, we used a single-molecule assay in which RNAP transcribes in the absence of CTP and therefore stalls at position +20 from the TSS, where it first needs to incorporate CTP (3,15). Because the footprint of elongating RNAP is approximately 25 base-pairs (22,23), stalling RNAP at +20 sterically prohibits a second RNAP from binding to the promoter. In the absence of accessory factors and for relatively low extending force (*F* ∼ 0.3 pN) and hence torque (|Γ| ∼ 12 pN·nm) the single, stalled RNAP remains bound indefinitely for both positively and negatively supercoiled DNA (Figure 1B and Supplementary Figure S1; see Table 4 for a summary of all supercoiling-based experiments) and as seen previously (15). Thus, we did not observe dissociation of RNAP from negatively supercoiled DNA over tens of hours (Supplementary Figure S1). However, by increasing the extending force to *F* ∼ 6 pN, and hence the torque applied to positively supercoiled DNA to Γ ∼ 52 pN·nm, we found that we could reliably observe RNAP dissociation on the scale of tens of minutes (Figure 1C). Because elevated negative torque denatures DNA, we could not probe the effect of higher torque in these conditions (Supplementary Figure S1).

**Figure 2.**
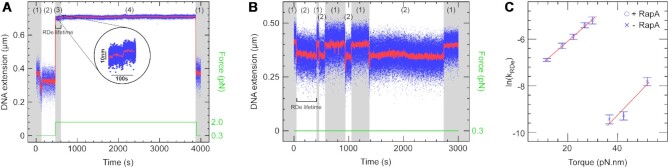
RapA removes stalled RNAP in a torque-sensitive manner. (**A**) Time-trace with RNAP, limiting NTPs and RapA in the ‘force-cycling’ assay. RNAP initiates and stalls on DNA. The force is increased and we observe rapid RNAP removal, which can be confirmed as the molecule extension returns to baseline upon returning to low force. (**B**) Time-trace with RNAP, limiting NTPs and RapA on positively supercoiled DNA in the low force ‘recycling assay’. RNAP initiates, stalls and is removed by RapA in an iterative cycle, with RDe lifetimes measured. (**C**) Plot of ln(k_RDe_) versus torque. RDe lifetimes were measured with and without RapA at relevant forces and fit according to a single-exponential (Supplementary Figure S3 and Supplementary Figure S2), with the mean values used here. 5 force points were taken with RapA and the gradient of the fit is 0.11 ± 0.02 rad/pN·nm, whereas 3 force points were taken without RapA and the gradient of the fit is 0.09 ± 0.01 rad/pN·nm.

To collect data on RNAP dissociation by positive torque, we therefore cycled positively supercoiled DNA between a low force state, in which RNAP can bind to and stall on DNA, and a high-force state, in which RNAP is forced to dissociate (Figure 1C). Because elevated positive torque inhibits transcription initiation (20), RNAP cannot re-initiate transcription until we return to the low-force state again. This ‘force-cycling’ assay allowed us to repeatedly measure the time that the stalled RNAP-DNA elongation complex (RDe) resides on DNA before dissociation (denoted as the RDe lifetime). RDe lifetimes follow single-exponential distributions (Supplementary Figure S2) from which a mean lifetime < *t*_RDe_ > could be obtained by fitting, yielding the rate of RNAP dissociation, *k*_RDe_ = 1/<*t*_RDe_ > . Next, because the torque, Γ, which acts on supercoiled DNA essentially scales with the square root of the extending force (see Materials and Methods), we repeated these measurements at three different extending forces to cover a range of torques. We obtain a linear relation between ln(*k*_RDe_) and Γ (Figure 2C), consistent with a simple Boltzmann law accounting for the system’s mechanical energy:

$$\begin{equation*}{{\rm{k}}_{{\rm{RDe}}}}\left( {\rm{\Gamma }} \right){\rm{ = }}{{\rm{k}}_{{\rm{RDe}}}}\left( {\rm{0}} \right){\rm{exp}}\left( {{\rm{\Gamma \theta /}}{{\rm{k}}_{\rm{B}}}{\rm{T}}} \right)\end{equation*}$$

where *k*(0) is the rate of dissociation at zero torque and θ is the extent of DNA rewinding in the transcription bubble that corresponds to the transition state intermediate to RNAP dissociation. To obtain *k*_RDe_(0) we extrapolate the line to zero torque, and this indicates that stalled RNAP remains bound to torsionally relaxed (unsupercoiled) DNA for close to 10^6^ s (∼12 days). Finally, the slope of the above line (0.11 ± 0.02 rad/pN·nm, Figure 2C) reflects the torque-sensitivity of RNAP dissociation, and from this analysis we obtain θ ∼ 22^o^, corresponding to rewinding of slightly less than one base-pair at the transition-state to RNAP dissociation. This suggests that the transcription bubble, although extremely stable against torque, is nevertheless ‘brittle’ in the sense that a small amount of rewinding can upset the entire structure.

### RapA removes stalled RNA polymerase in a torque-sensitive manner

To see how RapA affects RDe lifetime, we added RapA to the ‘force-cycling’ assay described above. We observed RapA to drastically shorten RDe lifetimes (Figure 2A), allowing us to observe dissociation of stalled elongation within a reasonable amount of time even at low levels of positive torque. In fact, it was even possible to observe repeated cycles of RNAP initiation/elongation/stalling followed by RapA-induced dissociation at a constant low force (0.3 and 0.7 pN, corresponding to torques of 12 and 18 pN·nm respectively, see Figure 2B; above ∼0.9 pN, RNAP could not reliably initiate transcription). RDe lifetimes were measured across five torque values and all followed single-exponential distributions (Supplementary Figure S3). The average of each lifetime distribution was obtained by fitting and plotted, along with RNAP-alone data, as ln(*k*_RDe_) versus torque (Figure 2C). RapA stimulates RNAP dissociation from positively supercoiled DNA and this process occurs faster under higher torque. Interestingly, we observe very similar gradients for the line fits of Figure 2C, comparing the previously-obtained value of θ/*k*_B_*T* = 0.11 ± 0.02 rad/pN·nm (θ ∼ 22^o^) in the absence of RapA to that of θ/*k*_B_*T* = 0.09 ± 0.01 rad/pN·nm (θ ∼ 18^o^) obtained in its presence. This indicates the underlying rearrangements which allow RNAP to finally dissociate from DNA are the same. Thus at zero-torque RapA activity releases RNAP in ∼2800 s, whereas in the absence of RapA release of RNAP occurs in ∼9 × 10^5^ s. Overall, RapA lowers the energy barrier between the RNAP-bound and RNAP-free states by ∼3.4 kcal/mol (∼5.8 *k*_B_*T*), accelerating the dissociation of RNAP from DNA.

### Observation of RNA polymerase removal by RapA on positively, but not negatively, supercoiled DNA

**Figure 3.**
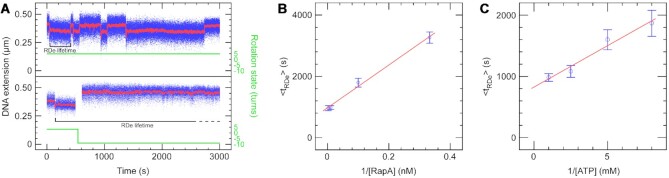
RapA removes RNA polymerase stalled on positively, but not negatively, supercoiled DNA. (**A**) Top panel: As shown in Figure 2b, time-trace showing the iterative stalling and removal of RNAP from positively supercoiled DNA in the presence of RapA. Bottom panel: Time-trace showing first the stalling of RNAP on positively supercoiled DNA, followed by a washing step to remove free RNAP and the addition of RapA and ATP (at ∼500 s), then the DNA was negatively supercoiled. RNAP remains stalled at +20 indefinitely. (**B**) Plot of < *t*_RDe_ > versus RapA concentration. RDe lifetimes were measured at various RapA concentrations and 1 mM ATP and fit according to a single-exponential (Supplementary Figure S8), with the mean values plotted here, giving a Michaelis constant of RapA for RNAP of *K*_m_ = 7.2 ± 0.7 nM and a rate-limiting step which proceeds at *k*_cat_ = 1.1 × 10^–3^ ± 0.5 × 10^–5^ s^–1^. (**C**) Plot of < *t*_RDe_ > versus ATP concentration. RDe lifetimes were measured at various ATP concentrations (but saturating RapA concentrations) and fit according to a single-exponential (Supplementary Figure S9), with the mean values plotted here. The *K*_m_ of RapA-RNAP for ATP is 160 ± 35 μM.

Following the observation that RapA enables removal of stalled RNAP from DNA at low torques, we next wanted to explore the relationship between RapA activity and the sign of DNA supercoiling. The initial ‘force-cycling’ assay could only be performed on positively supercoiled DNA as negatively supercoiled DNA forms denaturation bubbles at forces above ∼0.3 pN (corresponding to torques more negative than -12 pN·nm) that would interfere with our assays (24). However, the low force ‘recycling assay’ can be carried out on either positively or negatively supercoiled DNA. As previously mentioned, in the absence of RapA, RDe lifetime on either positively or negatively supercoiled DNA extended at ∼0.3 pN was consistently longer than the experimental time-frame (i.e. days). As Figure 2B and Figure 3A (top panel) show, RapA drastically shortens the RDe lifetime on positively supercoiled DNA. Strikingly, when the DNA was negatively supercoiled, RapA had no effect on RDe lifetime (Figure 3A, bottom panel). We were only able to observe RapA remove stalled RNAP from positively supercoiled DNA and never on negatively supercoiled DNA (Supplementary Figure S1).

### Kinetics of RNA polymerase removal by RapA

To interrogate the kinetics of RapA action as seen by analysing the RDe lifetime we used positively supercoiled DNA in the low force ‘recycling assay’ (Figure 3A, top panel). We measured RDe lifetime as a function of RapA (Figure 3B) and ATP concentrations (Figure 3C). The *K*_m_ of RapA for stalled RNAP is 7.2 ± 0.7 nM and the *K*_m_ of the RapA-RNAP complex for ATP is 160 ± 35 μM, generally in agreement with previously published bulk biochemical data (5). At saturating RapA and ATP concentration, a rather slow rate-limiting step is observed: *k*_cat_ = 1.1 × 10^–3^ ± 0.5 × 10^–5^ s^–1^ for dissociation of RNAP from the substrate. As previously mentioned RapA and ATP alone (no RNAP) on positively supercoiled DNA gave rise to rare and very short-lived increases in DNA extension (Supplementary Figure S4) indicating potentially weak over-winding or right-handed wrapping of DNA.

### RapA is functionally inhibited by GreB

**Figure 4.**
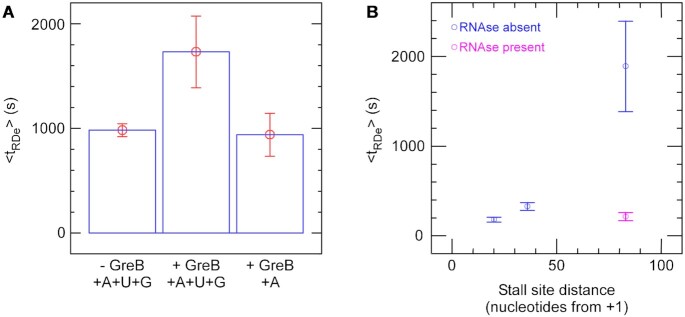
RapA removes RNAP by backtracking. (**A**) GreB slows RapA-mediated RNAP removal. Using the low force ‘recycling assay’, RapA removes 97% of stalled RNAP with a < *t*_RDe_ > of 983 ± 62s. In the presence of GreB, RapA removes 52% of stalled RNAP with a < *t*_RDe_ > of 1732 ± 343s. In the presence of GreB but absence of UTP and GTP, RapA removes 84% of stalled RNAP with a < *t*_RDe_ > of 940 ± 205 s. (**B**) Plot of < t_RDe_ > versus stall site distance from promoter. RDe lifetimes were measured with RNAP, limiting NTPs, saturating RapA and saturating ATP in the ‘force-cycling’ assay. These lifetimes were fit according to a single-exponential (Supplementary Figure S10), with the mean values shown here. As the distance between the stall site and the promoter increases, the longer it takes RapA to remove the RNAP, unless RNAse A is present (shown in magenta).

In order to understand the mechanism by which RapA removes RNAP from DNA, we sought to observe how the addition of the anti-backtracking factor GreB may affect RapA activity. If RapA removes RNAP by backtracking, then GreB should counteract it because GreB can reactivate backtracked RNAP, enabling it to elongate down to the stall site again (25,26). When we thus added 50nM GreB to the low force ‘recycling assay,’ we observed that roughly 50% of RNAP remained bound indefinitely, as if RapA activity were lost. This is to be compared to the removal of nearly 100% of RNAP in a comparable timeframe in the absence of GreB (Table 1). For those RNAP molecules that are removed by RapA in the presence of GreB, <*t*_RDe_ > is roughly doubled (Figure 4A and Supplementary Figure S5). As structural data shows these proteins do not share or overlap binding sites on RNAP (8,27), this is strong evidence to indicate GreB functionally opposes the action of RapA. Tellingly, when we omit UTP and GTP (by washing out free NTPs once RNAP is stalled, before adding just RapA, ATP and GreB), <*t*_RDe_ > is comparable to that observed absent GreB (Table 1, Figure 4A and Supplementary Figure S5), indicating the inhibitory effect of GreB is lost. Thus, the presence of the full complement of NTPs needed to support transcription is required for the inhibitory effect of GreB to be observed, supporting a backtracking mechanism of action for RapA-mediated removal of stalled RNAP.

**Table 1.** GreB inhibits RNAP removal by RapA. The addition of 50 nM GreB roughly halves the number of RNAP acted on by RapA and roughly doubles the < *t*_RDe_ > for those which are removed by RapA. The subsequent omission of UTP and GTP gives a < *t*_RDe_ > similar to that observed when GreB is absent, showing that NTPs must be present for GreB to exert its inhibitory effect

[GreB] (nM)	[ATP] (mM)	[UTP] (μM)	[GTP] (μM)	Number of molecules with RNA polymerase initiation	Number of molecules with RNA polymerase removed	Percentage of RNA polymerase removed by RapA (%)	<*t*_RDe_> (s)
0	1	200	200	74	72	97	983 ± 62
50	1	200	200	87	45	52	1732 ± 343
50	1	0	0	43	36	84	940 ± 205

### RapA takes longer to remove RNA polymerase stalled further away from the promoter

To further test the backtracking model, we designed DNA constructs with stall sites at different distances from the promoter. If RapA removes RNAP by backtracking, then RDe lifetime should increase as we increase the distance RNAP must backtrack to be dissociated. To test this, we built two additional DNA constructs, one with a stall site at +36 from the TSS and one with a stall site at +83 from the TSS. Whereas the former construct uses NTP starvation to stall RNAP at +36, the latter DNA construct uses NTP starvation to stall the RNAP at +15, following which we wash out all RNAP in solution and add in all four NTPs (and RapA) which allows RNAP to rapidly transcribe up to a stall-inducing cyclopyrimidine dimer (CPD) situated at +83. It has been shown previously that a CPD also irreversibly stalls RNAP indefinitely (15) and we have shown that RapA action on RNAP is independent of stall cause (Supplementary Figure S6). We compared < *t*_RDe_ > with each DNA construct and observe that RapA takes longer to remove RNAP stalled further away from the promoter (Figure 4B). Importantly, when RNAP is stalled at +83 and RNAse A is added in addition to RapA and ATP, <*t*_RDe_ > is drastically reduced (Figure 4B), comparable to that observed with stalling at +20. In other words, when the RNA length is shortened, RapA removes RNAP faster.

In further support of RNAP backtracking prior to dissociation, we observe a drastic loss of RapA-mediated RNAP dissociation when an oligonucleotide is hybridized to the nascent RNA. On the construct stalling RNAP at +36 from the TSS, hybridisation of a 17 base-pair ssDNA oligonucleotide to the 5′ end of the nascent RNA inhibited RapA action. Of 22 stalled RNAPs, only 4 were removed by RapA over 5 h (18%; compared to 81% of stalled RNAP removed by RapA over 50 min normally; see Table 2). This indicates that a DNA:RNA hybrid upstream of RNAP impedes RNAP dissociation from DNA.

**Table 2.** Hybridising an oligonucleotide to the nascent RNA inhibits RNAP removal by RapA

[Hybridising oligonucleotide] (nM)	Number of molecules with RNA polymerase initiation	Number of molecules with RNA polymerase removed	Percentage of RNA polymerase removed by RapA (%)	<*t*_RDe_> (s)
0	105	85	81 (within 1 h)	328 ± 43.4
100	22	4	18 (within 5 h)	4070 ± 2700

### Direct observation of backtracking and RNA polymerase dissociation using a tethered-RNA polymerase assay

To directly observe RNAP dynamics in the presence of RapA at zero supercoiling, we used a bead-tethered-RNAP assay (‘tethered RNAP’) (14). In this assay, biotinylated-RNAP is stalled at +20, using NTP starvation, from a promoter located at one end of an ∼8 kb DNA construct (see Supplementary Figure S7a) (14). The biotinylated-RNAP is then tethered to a streptavidin-coated magnetic bead and the linear digoxigenin-labeled DNA construct is anchored to the anti-digoxigenin-coated flow cell surface. Under a 1pN force, the bead, which now informs us on the position of RNAP along the DNA, is held ∼2 μm from the surface. Firstly, we wanted to see if RapA could dissociate stalled RNAP from DNA at zero supercoiling. We added RapA and ATP to assembled complexes and observed that 80% RNAP were removed within 6 h with an average lifetime of 3060 s ± 233 (Supplementary Figure S7b and Table 3). This is in comparison to 0% RNAP removal over 19 h when RapA and ATP were omitted (Table 3).

**Table 3.** Tethered-RNA polymerase experiment summary

Condition	No. of molecules	Molecules removed from DNA (%)	Molecules showing backtracking (%)	Of these, molecules backtracking to the promoter (%)	<*t*_RDe_> (s)	Experiment duration (h)
Tethered-RNAP stalled at +20 (RapA absent)	6	0	n/a	n/a	n/a	19
Tethered-RNAP stalled at +20 in presence of RapA and ATP	10	80	n/a	n/a	3060 ± 233	6
Tethered-RNAP stalled hundreds of base-pairs from promoter in presence of RapA and ATP	18	67	67	33	10,100 ± 1000	6

**Table 4.** Overview of all supercoiling-based experiments performed

Stall site distance from +1	DNA supercoiling status	Force (pN)	Torque (pN.nm)	[RapA]	[ATP]	[Additional component]	Stalling cause	<*t*_RDe_> (s)	Events (*n*)	Where to find
+20	Positive	0.3	12	-	1 mM	-	C-less cassette	-	-	Figure 1B
+20	Negative	0.3	-12	-	1 mM	-	C-less cassette	-	-	Supplementary Figure S1
+20	Positive	3.0	37	-	1 mM	-	C-less cassette	12600 ± 2430	61	Supplementary Figure S2a
+20	Positive	4.0	42	-	1 mM	-	C-less cassette	10800 ± 1920	73	Supplementary Figure S2b
+20	Positive	6.0	52	-	1 mM	-	C-less cassette	2500 ± 426	64	Supplementary Figure S2c and Figure 1C (time-trace)
+20	Positive	0.3	12	100 nM	1 mM	-	C-less cassette	983 ± 61.9	289	Figure 3B, Figure 3C, Supplementary Figure S8b, Supplementary Figure S3a, Supplementary Figure S9a and Figure 2B (time-trace)
+20	Positive	0.7	18	100 nM	1 mM	-	C-less cassette	542 ± 63.9	112	Supplementary Figure S3b
+20	Positive	1.1	22	100 nM	1 mM	-	C-less cassette	365 ± 41.6	150	Supplementary Figure S3c
+20	Positive	1.6	27	100 nM	1 mM	-	C-less cassette	223 ± 23.9	248	Supplementary Figure S3d
+20	Positive	2.0	30	100 nM	1 mM	-	C-less cassette	181 ± 27.5	87	Supplementary Figure S3e and Figure 2A (time-trace)
+20	Negative	0.3	-12	100 nM	1 mM	-	C-less cassette	-	-	Figure 3B bottom panel (time-trace)
+20	Positive	0.3	12	500 nM	1 mM	-	C-less cassette	965 ± 82.5	212	Figure 3B and Supplementary Figure S8a
+20	Positive	0.3	12	10 nM	1 mM	-	C-less cassette	1800 ± 144	244	Figure 3B and Supplementary Figure S8c
+20	Positive	0.3	12	3 nM	1 mM	-	C-less cassette	3270 ± 182	421	Figure 3B and Supplementary Figure S8d
+20	Positive	0.3	12	100 nM	400 μM	-	C-less cassette	1080 ± 97.1	195	Figure 3C and Supplementary Figure S9b
+20	Positive	0.3	12	100 nM	200 μM	-	C-less cassette	1600 ± 163	249	Figure 3C and Supplementary Figure S9c
+20	Positive	0.3	12	100 nM	125 μM	-	C-less cassette	1870 ± 212	135	Figure 3C and Supplementary Figure S9d
+20	Positive	0.3	12	100 nM	1 mM	50 nM GreB	C-less cassette	1730 ± 343	45	Figure 4A, Supplementary Figure S5 and Table 1
+20	Positive	0.3	12	100 nM	1 mM	50 nM GreB (no UTP or GTP)	C-less cassette	940 ± 205	36	Figure 4A, Supplementary Figure S5 and Table 1
+20	Positive	2.0	30	100 nM	1 mM	-	CPD	796 ± 179	37	Supplementary Figure S6
+20	Positive	2.0	30	100 nM	1 mM	-	C-less cassette	181 ± 27.5	87	Figure 4B and Supplementary Figure S10a
+36	Positive	2.0	30	100 nM	1 mM	-	C-less cassette	328 ± 43.4	85	Figure 4B and Supplementary Figure S10b
+36	Positive	2.0	30	100 nM	1 mM	100nM RNA-hybridising oligo	C-less cassette	4070 ± 2700	4	Table 2
+83	Positive	2.0	30	100 nM	1 mM	-	CPD	1890 ± 505	24	Figure 4B and Supplementary Figure S10c
+83	Positive	2.0	30	100 nM	1 mM	100 μg/ml RNAse A	CPD	215 ± 45.5	38	Figure 4B and Supplementary Figure S10d

(unless stated otherwise, [GTP] = [UTP] = 200 μM, and temperature = 34°C).

We then sought to directly observe backtracking prior to RNAP removal. However, since backtracking over only 20 base-pairs is difficult to detect (at 1 pN this corresponds to bead movement on the order of 5 nm), we next observed the action of RapA on RNAP having transcribed several hundred bases. RNAP transcription resulted in clear bead motion toward the surface (Supplementary Figure S7c and d). By making RNAP transcribe in the presence of RapA, motion of the bead towards the surface was interrupted and reversed via backtracking in 67% of molecules, and all molecules that backtracked dissociated from the DNA (Supplementary Figure S7d and Table 3). Some 33% of these molecules backtracked all the way to the promoter, as observed by upwards bead motion back to the baseline height. We note that a majority of RNAP dissociate prior to backtracking all the way to the promoter. These tethered-RNAP experiments show us that RapA can remove stalled RNAP from DNA at zero supercoiling, and that RapA mediates backtracking of RNAP that ultimately ends in dissociation.

## DISCUSSION

Many studies have shown how accessory factors may activate or remodel RNAP at various stages of transcription. In particular, many factors can act on RNAP that is paused or stalled in elongation phase, however little is known about the intrinsic stability of the RNAP-RNA-DNA complex alone. Indeed, we have never observed stalled RNAP to dissociate from DNA at the low forces typically used for single-molecule transcription assays. However, at higher force, thus higher torque, we have observed the dissociation of stalled RNAP from DNA in the absence of accessory factors.

We have shown that RapA accelerates the dissociation of stalled RNAP from DNA. Four main lines of evidence point towards a mechanism involving backtracking. First, RapA is functionally inhibited by the anti-backtracking factor GreB (and this inhibition is ablated when NTPs are omitted, confirming inhibition is not purely via steric competition); secondly, increasing the stall site distance from the promoter, thus increasing the length of the RNA that must be threaded back through the RNAP, increases the time taken for RapA to remove RNAP (unless the RNA is degraded by RNAse A, in which case RapA removes RNAP relatively faster); thirdly, the hybridization of an oligonucleotide to the nascent RNA inhibits RNAP dissociation; and finally, using tethered-RNAP we directly observe RapA-mediated backtracking followed by RNAP dissociation.

We note that < *t*_RDe_ > increases faster than linearly with increasing stall site distance from the promoter. If RNAP were to be removed via processive backtracking by a single RapA, i.e. via a succession of irreversible steps, one would expect < *t*_RDe_ > to increase linearly with distance. The observed non-linearity could be explained by a model wherein RapA only backtracks stalled RNAP by one base-pair, allowing another slower process to complete the backtracking. For example, backtracked RNAP is known to undergo a random walk, and this could give rise to non-linear time-dependencies (28). Therefore, one hypothesis is that in these experiments RNAP dissociation occurs by a first RapA-mediated backtracking step followed by random diffusion of the un-ratcheted motor until the entire RNA is threaded back through the RNAP, and RNAP dissociates from the DNA.

**Figure 5.**
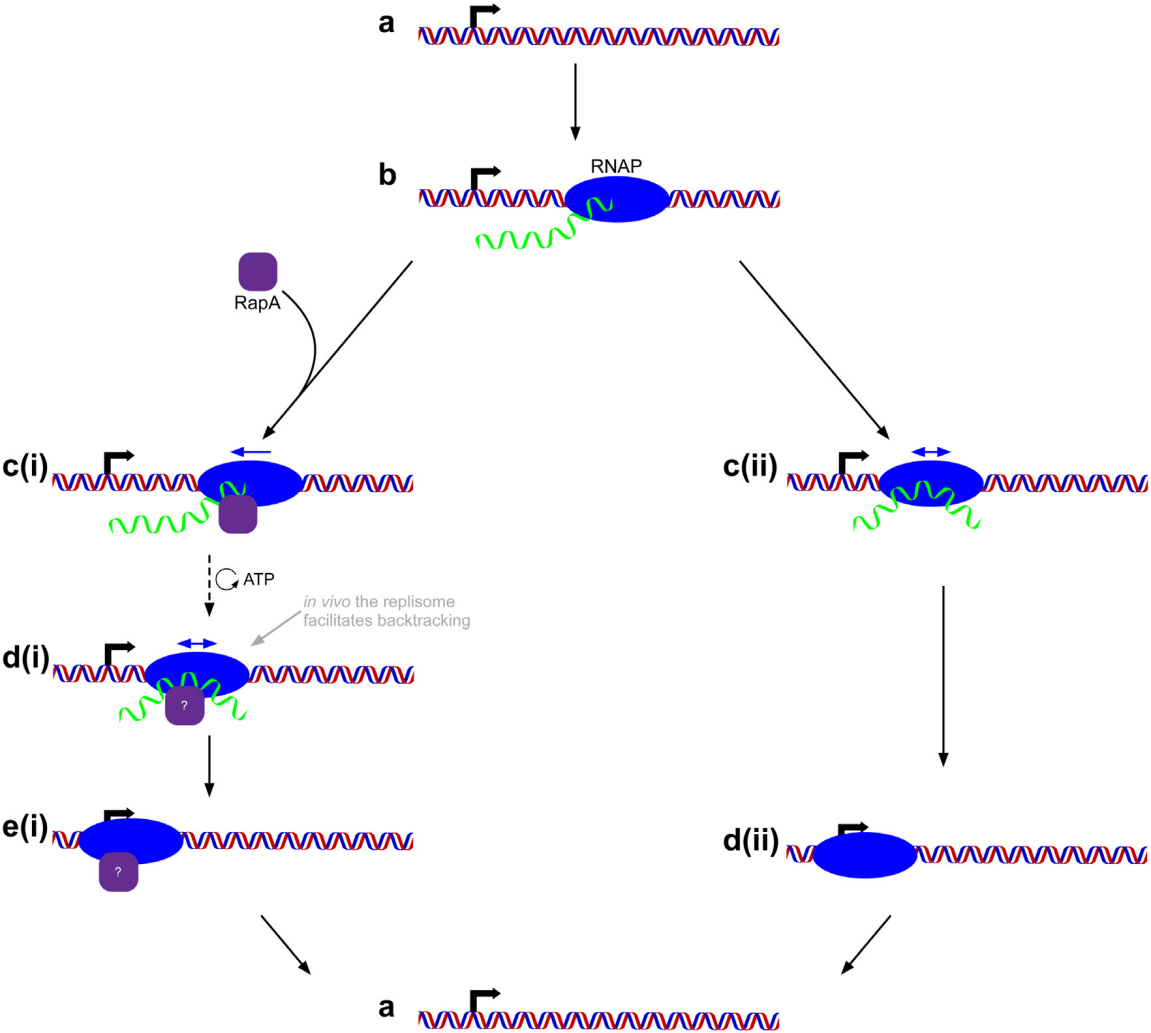
Model of stalled RNAP dissociation from positively supercoiled DNA. RNAP transcribes on positively supercoiled DNA (a) and becomes stalled (b). In the presence of RapA (left branch), RapA first mediates the ATP-dependent backtracking of RNAP (c(i) to d(i)) until RNAP is freely diffusing (we note here that *in vivo* other motors such as the replisome would greatly bias backtracking at this point). Once RNAP has diffused (or, *in vivo*, been rapidly pushed back) to the promoter (d(i) to e(i)), the RNA is released and RNAP dissociates from the DNA (e(i) to a). We estimate this process, at zero force and absent further accessory factors, to take on the order of 10^3^ s. In the absence of RapA but under high positive torque (right branch), RNAP backtracks slowly to the promoter (c(ii) to d(ii)). The RNA is released and RNAP dissociates from the DNA (d(ii) to a). We estimate this process, at zero force, to take on the order of 10^6^ s. The promoter is now free to be bound by another RNAP.

Importantly, we propose that *in vivo* the first RapA-mediated backtracking step is followed by active backtracking by another molecular motor, such as DNA polymerase in the case of head-on conflict between DNA and RNA polymerase. We note that in this scenario the DNA on which RNAP is located would tend to be positively supercoiled. This RapA-mediated RNAP dissociation is graphically depicted and compared to RapA-independent RNAP dissociation in the model in Figure 5.

We only observe RapA-mediated RNAP dissociation on DNA that is positively supercoiled and have never observed RapA removing RNAP from negatively supercoiled DNA. Our observation of RapA-mediated RNAP dissociation from linear, non-supercoiled DNA suggests that RapA could potentially also function on negatively supercoiled DNA, although it is important to remember the assays performed on positively supercoiled and non-supercoiled DNA are distinct and a 1 pN force acts in the direction opposing transcription in the tethered-RNAP assay. Of course on negatively supercoiled DNA there is an energetic penalty to ejecting RNAP and rewinding shut the transcription bubble, whereas on positively supercoiled DNA there is an energetic advantage to ejecting RNAP and rewinding shut the transcription bubble. However, extrapolating the ln(*k*_RDe_) versus torque curve (Figure 2C) to the negative torque value (Γ ∼ -12 pN·nm) at which experiments were conducted indicates that, under simplifying assumptions, RNAP should have been dissociated on experimentally accessible timescales similar to the slower ones observed absent RapA. That this is not the case suggests that the transcription bubble, embedded in the asymmetric RNAP structure, experiences different energy landscapes to rewinding under positive and negative supercoiling. It will be interesting in the future to determine therefore whether or not the structure of the transcription elongation complex is altered by the sign of the DNA’s supercoiling.

Of course this inability to displace RNAP from negatively supercoiled DNA could be physiologically important for RapA given that the bacterial nucleoid is generally negatively supercoiled and this could lead RapA to disrupt transcriptional programs. Positive supercoiling is generated only in specific contexts such as in front of the replication fork or within highly transcribed genes, and particularly when these contexts are coincident. For instance, RapA may act to remove RNAPs that come to be stalled due to the positive torque they experience in front of the replisome. Indeed, DNA is known to be positively supercoiled in front of the replication fork (29), and positive torque of ∼18 pN.nm in front of RNAP is known to stall RNAP (30). In these conditions that same positive torque could allow RapA to rapidly backtrack RNAP by one base-pair in a manner specific to these loci. This would inactivate the transcription elongation complex and thus allow the replisome to chase the un-ratcheted RNAP off the 5′ end of its RNA in just a few seconds, as described above. Therefore, we propose that at least one of the main biological roles of RapA is the resolution of motor conflicts, by biasing RNAP backtracking, allowing the opposing motor to ‘win’ the battle.

## DATA AVAILABILITY

All data files are available upon reasonable requests.

## Supplementary Material

gkac558_Supplemental_File

## Notes

Present address: James Portman, Department of Biological Chemistry and Molecular Pharmacology, Harvard Medical School, Boston MA 02115, USA.

## References

[1] Chakraborty A. , Wang D., Ebright Y.W., Korlann Y., Kortkhonjia E., Kim T., Chowdhury S., Wigneshweraraj S., Irschik H., Jansen R. et al. Opening and closing of the bacterial RNA polymerase clamp. Science. 2012; 337:591–595. 22859489 10.1126/science.1218716 PMC3626110

[2] Park J.S. , Marr M.T., Roberts J.W. E. coli transcription repair coupling factor (mfd protein) rescues arrested complexes by promoting forward translocation. Cell. 2002; 109:757–767. 12086674 10.1016/s0092-8674(02)00769-9

[3] Fan J. , Leroux-Coyau M., Savery N.J., Strick T.R. Reconstruction of bacterial transcription-coupled repair at single-molecule resolution. Nature. 2016; 536:234–237. 27487215 10.1038/nature19080

[4] Portman J.R. , Brouwer G.M., Bollins J., Savery N.J., Strick T.R. Cotranscriptional R-loop formation by mfd involves topological partitioning of DNA. Proc. Natl. Acad. Sci. 2021; 118:e2019630118. 33827922 10.1073/pnas.2019630118 PMC8053947

[5] Sukhodolets M.V. , Jin D.J. RapA, a novel RNA polymerase-associated protein, is a bacterial homolog of SWI2/SNF2. J. Biol. Chem. 1998; 273:7018–7023. 9507009 10.1074/jbc.273.12.7018

[6] Burgess R.R. , Travers A.A., Dunn J.J., Bautz E.K.F. Factor stimulating transcription by RNA polymerase. Nature. 1969; 221:43–46. 4882047 10.1038/221043a0

[7] Muzzin O. , Campbell E.A., Xia L., Severinova E., Darst S.A., Severinov K. Disruption of escherichia coli hepA, an RNA polymerase-associated protein, causes UV sensitivity. J. Biol. Chem. 1998; 273:15157–15161. 9614128 10.1074/jbc.273.24.15157

[8] Liu B. , Zuo Y., Steitz T.A. Structural basis for transcription reactivation by RapA. Proc. Natl. Acad. Sci. U. S. A. 2015; 112:2006–2010. 25646438 10.1073/pnas.1417152112 PMC4343176

[9] McKinley B.A. , Sukhodolets M.V. Escherichia coli RNA polymerase-associated SWI/SNF protein RapA: evidence for RNA-directed binding and remodeling activity. Nucleic Acids Res. 2007; 35:7044–7060. 17913745 10.1093/nar/gkm747 PMC2175355

[10] Sukhodolets M.V. , Jin D.J. Interaction between RNA polymerase and RapA, a bacterial homolog of the SWI/SNF protein family. J. Biol. Chem. 2000; 275:22090–22097. 10801781 10.1074/jbc.M000056200

[11] Sukhodolets M.V. , Cabrera J.E., Zhi H., Jin D.J. RapA, a bacterial homolog of SWI2/SNF2, stimulates RNA polymerase recycling in transcription. Genes Dev. 2001; 15:3330–3341. 11751638 10.1101/gad.936701 PMC312849

[12] Kakar S. , Fang X., Lubkowska L., Zhou Y.N., Shaw G.X., Wang Y.X., Jin D.J., Kashlev M., Ji X. Allosteric activation of bacterial swi2/snf2 (Switch/Sucrose Non-fermentable) protein RapA by RNA polymerase. Biochem. Struc. Stud.*. 2015; 290:23656–23669. 10.1074/jbc.M114.618801 PMC4583045 26272746

[13] Richmond M. , Pasupula R.R., Kansara S.G., Autery J.P., Monk B.M., Sukhodolets M.V. RapA, escherichia coli RNA polymerase SWI/SNF subunit-dependent polyadenylation of RNA. Biochemistry. 2011; 50:2298–2312. 21299217 10.1021/bi101017x

[14] Graves E.T. , Duboc C., Fan J., Stransky F., Leroux-Coyau M., Strick T.R. A dynamic DNA-repair complex observed by correlative single-molecule nanomanipulation and fluorescence. Nat. Struct. Mol. Biol. 2015; 22:452–457. 25961799 10.1038/nsmb.3019

[15] Howan K. , Smith A.J., Westblade L.F., Joly N., Grange W., Zorman S., Darst S.A., Savery N.J., Strick T.R. Initiation of transcription-coupled repair characterized at single-molecule resolution. Nature. 2012; 490:431–434. 22960746 10.1038/nature11430 PMC3475728

[16] Qayyum M.Z. , Molodtsov V., Renda A., Murakami K.S. Structural basis of RNA polymerase recycling by the swi2/snf2 family of ATPase RapA in escherichia coli. J. Biol. Chem. 2021; 297:101404. 34774797 10.1016/j.jbc.2021.101404 PMC8666675

[17] Shaw G. , Gan J., Zhou Y.N., Zhi H., Subburaman P., Zhang R., Joachimiak A., Jin D.J., Ji X. Structure of RapA, a swi2/snf2 protein that recycles RNA polymerase during transcription. Structure. 2008; 16:1417–1427. 18786404 10.1016/j.str.2008.06.012 PMC2607195

[18] Duboc C. , Fan J., Graves E.T., Strick T.R. Preparation of DNA substrates and functionalized glass surfaces for correlative nanomanipulation and colocalization (NanoCOSM) of single molecules. Methods Enzymol. 2017; 582:275–296. 28062038 10.1016/bs.mie.2016.09.048

[19] Strick T.R. , Allemand J.-F., Bensimon D., Bensimon A., Croquette V. The elasticity of a single supercoiled DNA molecule. Science. 1996; 271:1835–1837. 8596951 10.1126/science.271.5257.1835

[20] Revyakin A. , Ebright R.H., Strick T.R. Promoter unwinding and promoter clearance by RNA polymerase: detection by single-molecule DNA nanomanipulation. Proc. Natl. Acad. Sci. 2004; 101:4776–4780. 15037753 10.1073/pnas.0307241101 PMC387324

[21] Strick T.R. , Allemand J.-F., Bensimon D., Croquette V. Stress-Induced structural transitions in DNA and proteins. Annu. Rev. Biophys. Biomol. Struct. 2000; 29:523–543. 10940258 10.1146/annurev.biophys.29.1.523

[22] Krummel B. , Chamberlin M.J. Structural analysis of ternary complexes of escherichia coli RNA polymerase. J. Mol. Biol. 1992; 225:239–250. 1593619 10.1016/0022-2836(92)90918-a

[23] Polyakov A. , Severinova E., Darst S.A. Three-dimensional structure of e. coil core RNA polymerase: promoter binding and elongation conformations of the enzyme. Cell. 1995; 83:365–373. 8521466 10.1016/0092-8674(95)90114-0

[24] Strick T.R. , Croquette V., Bensimon D. Homologous pairing in stretched supercoiled DNA. Proc. Natl. Acad. Sci. U. S. A. 1998; 95:10579–10583. 9724746 10.1073/pnas.95.18.10579 PMC27937

[25] Borukhov S. , Sagitov V., Goldfarb A. Transcript cleavage factors from E. coli. Cell. 1993; 72:459–466. 8431948 10.1016/0092-8674(93)90121-6

[26] Toulme F. , Mosrin-Huaman C., Sparkowski J., Das A., Leng M., Rahmouni A.R. GreA and GreB proteins revive backtracked RNA polymerase in vivo by promoting transcript trimming. EMBO J. 2000; 19:6853–6859. 11118220 10.1093/emboj/19.24.6853 PMC305891

[27] Opalka N. , Chlenov M., Chacon P., Rice W.J., Wriggers W., Darst S.A. Structure and function of the transcription elongation factor GreB bound to bacterial RNA polymerase. Cell. 2003; 114:335–345. 12914698 10.1016/s0092-8674(03)00600-7

[28] Galburt E.A. , Grill S.W., Wiedmann A., Lubkowska L., Choy J., Nogales E., Kashlev M., Bustamante C. Backtracking determines the force sensitivity of RNAP II in a factor-dependent manner. Nature. 2007; 446:820–823. 17361130 10.1038/nature05701

[29] Hiasa H. , Marians K.J. Two distinct modes of strand unlinking during theta-type DNA replication. J. Biol. Chem. 1996; 271:21529–21535. 8702938 10.1074/jbc.271.35.21529

[30] Ma J. , Tan C., Gao X., Fulbright R.M. Jr, Roberts J.W., Wang M.D. Transcription factor regulation of RNA polymerase's torque generation capacity. Proc Natl Acad Sci U S A. 2019; 116:2583–2588. 30635423 10.1073/pnas.1807031116 PMC6377492

