# Supplementary material for: On the stability of stalled RNA polymerase and its removal by RapA
Source: Nucleic Acids Res. 2022 Jul 12;50(13):7396–405. doi: 10.1093/nar/gkac558 (PMC9303389; doi:10.1093/nar/gkac558)
Supplement: gkac558_Supplemental_File [file gkac558_supplemental_file.docx]

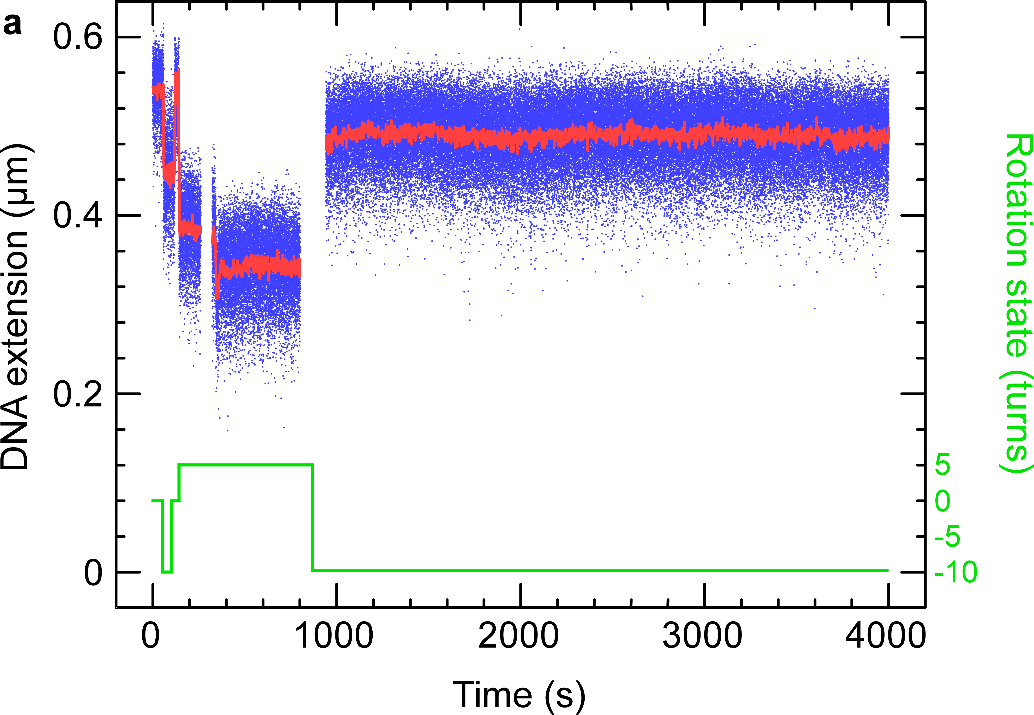


**Supplementary Figure 1:** Time-trace with RNAP and limiting NTPs on negatively supercoiled DNA. Components are injected (~300s) and RNAP stalls on positively supercoiled DNA. The DNA is then negatively supercoiled (~900s). The difference is DNA extension at the -10 rotation state confirms RNAP remained stalled on DNA during the negative supercoiling. Across 40 molecules over 20 hours all RNAP molecules remained bound to DNA.


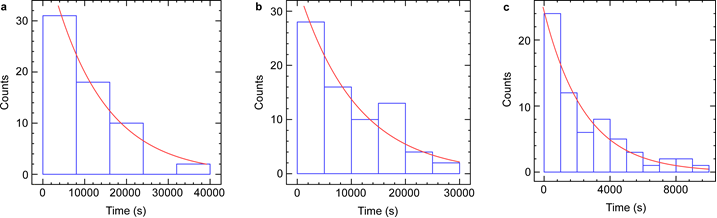


**Supplementary Figure 2**: RDe lifetime distributions at different forces without RapA. **a)** Histogram of RDe lifetimes measured at 3.0pN force (n = 61; average = 12590s ± 2430). **b)** Histogram of RDe lifetimes measured at 4.0pN force (n = 73; average = 10810s ± 1920). **c)** Histogram of RDe lifetimes measured at 6.0pN force (n = 64; average = 2500s ± 426).


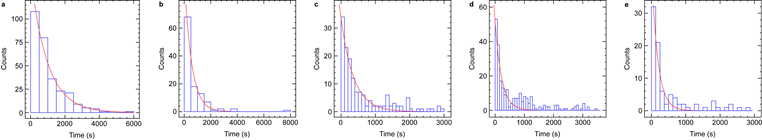


**Supplementary Figure 3:** RDe lifetime distributions at different forces with RapA. **a)** Histogram of RDe lifetimes measured at 0.3pN force (n = 289; average = 983s ± 61.9). **b)** Histogram of RDe lifetimes measured at 0.7pN force (n = 112; average = 542s ± 63.9). **c)** Histogram of RDe lifetimes measured at 1.1pN force (n = 150; average = 365s ± 41.6). **d)** Histogram of RDe lifetimes measured at 1.6pN force (n = 248; average = 223s ± 23.9). **e)** Histogram of RDe lifetimes measured at 2.0pN force (n = 87; average = 181s ± 27.5).


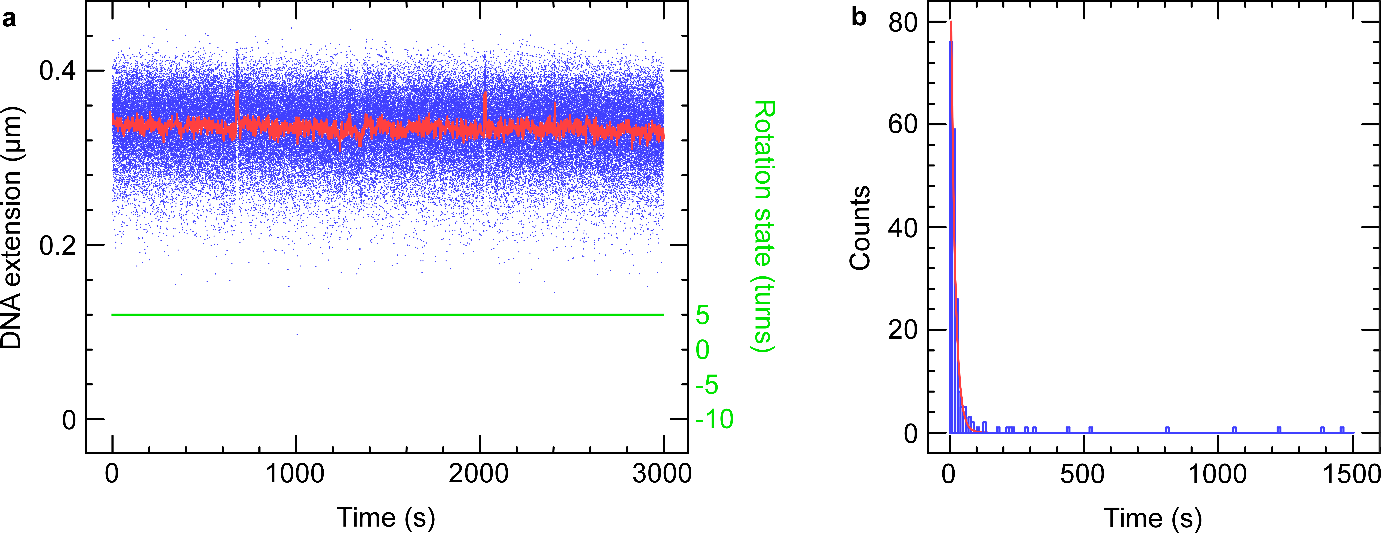


**Supplementary Figure 4**: RapA and ATP alone on DNA. **a)** Time-trace on positively supercoiled DNA with 100nM RapA and 1mM ATP. DNA extension increase events are seen at ~700s and ~2000s. **b)** Histogram of these event lifetimes (n = 205; average = 15.5s ± 1.24).


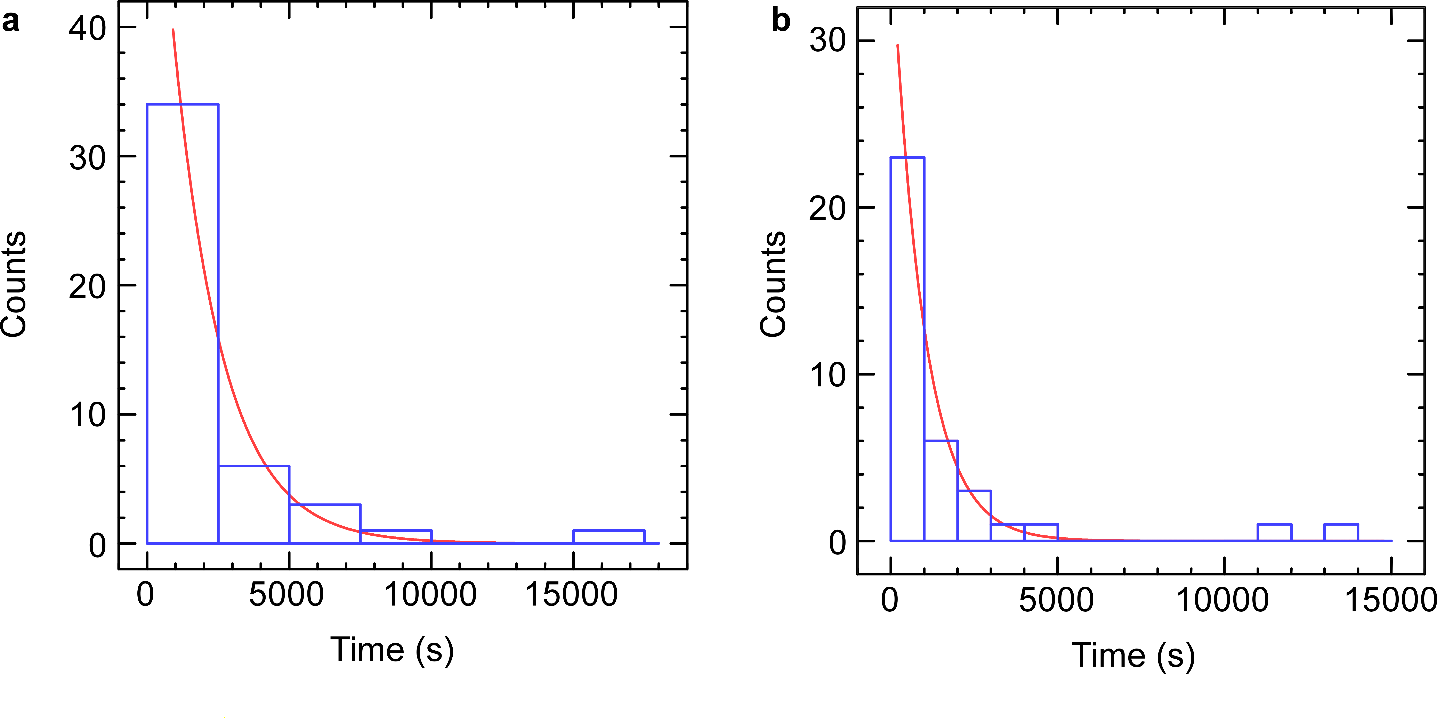
**Supplementary Figure 5**: RDe lifetime distribution with 50nM GreB. **a)** Histogram of RDe lifetimes measured in the low force ‘recycling assay’ (0.3pN; saturating ATP and RapA concentration; [UTP] = [GTP] = 200µM) with 50nM GreB present (n = 45; average = 1732s ± 343). **b)** Histogram of RDe lifetimes measured in the low force ‘recycling assay’ (0.3pN; saturating ATP and RapA concentration; no UTP or GTP) with 50nM GreB present (n = 36; average = 940s ± 205).

**
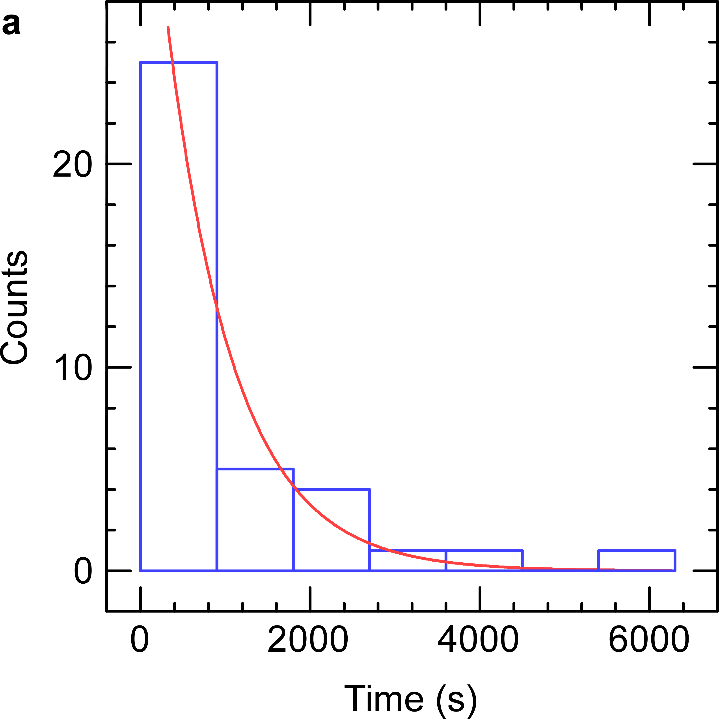
**

**Supplementary Figure 6**: RDe lifetime distribution with CPD stalling. **a)** Histogram of RDe lifetimes measured in the ‘force-cycling’ assay (2pN; saturating ATP and RapA concentration) using a CPD to stall RNAP at +20 (n = 37; average = 796s ± 179). This is not significantly different from the average RDe lifetime obtained under the same conditions but with NTP starvation as the stalling cause.

**
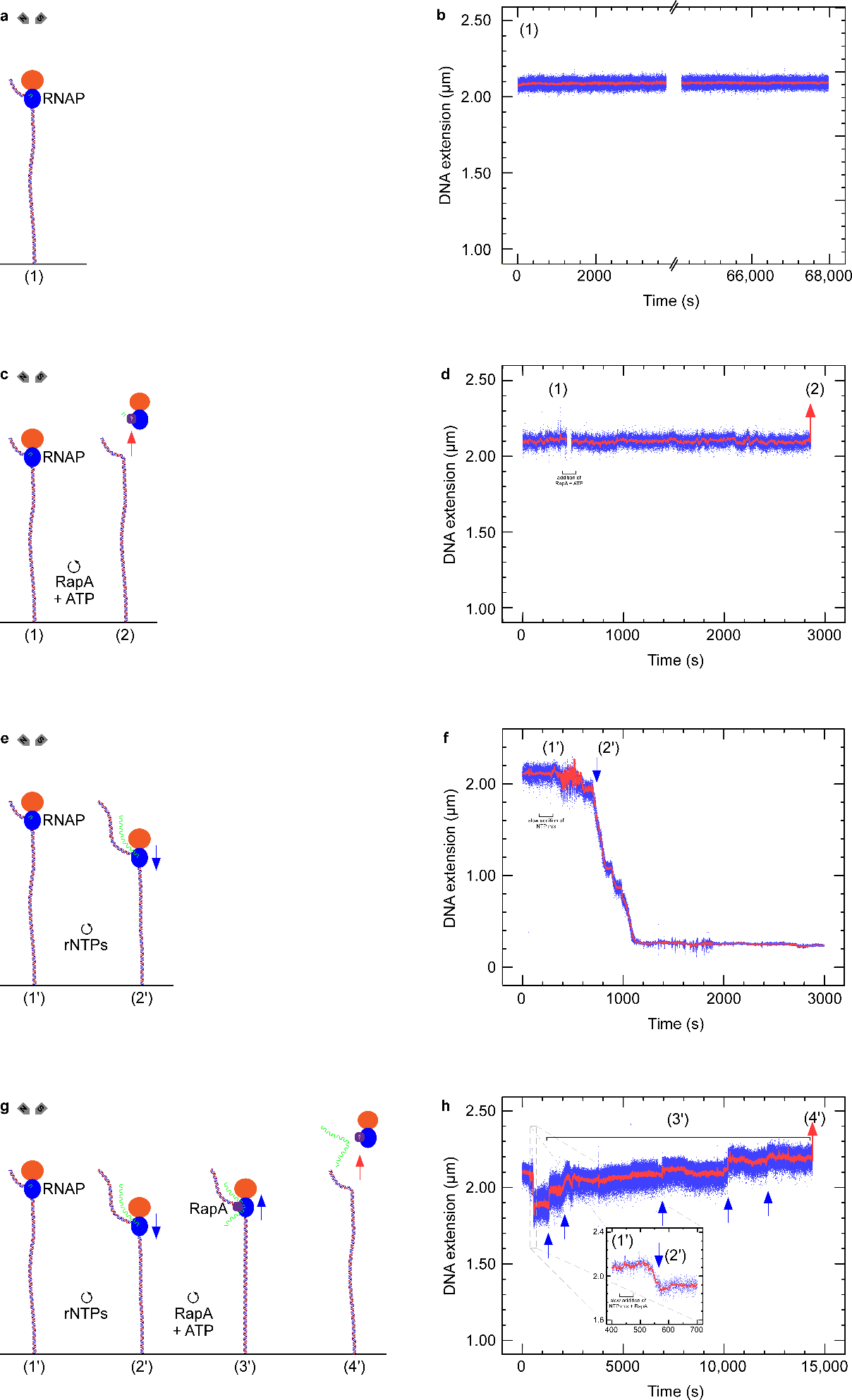
**

**Supplementary Figure 7**: Tethered-RNA polymerase experiments. **a)** and **b)** Model and time-trace, respectively, showing tethered-RNAP stalled at +20 (1) absent RapA and ATP. **c)** and **d)** Model and time-trace, respectively, showing tethered-RNAP stalled at +20 (1) followed by the addition of RapA and ATP (~500s), causing RNAP to dissociate and the bead to disappear (2). **e)** and **f)** Model and time-trace, respectively, showing tethered-RNAP (1’) transcribe towards the surface (2’). **g)** and **h)** Model and time-trace, respectively, showing tethered-RNAP (1’) transcribe towards the surface (2’) before stalling and RapA-dependent backtracking (3’) causing RNAP to ultimately dissociate and the bead to disappear (4’).

**
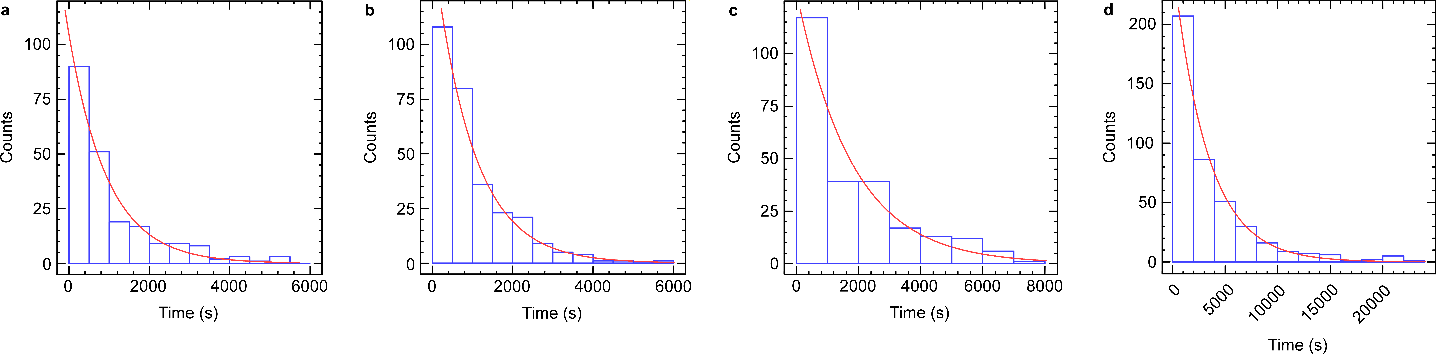
Supplementary Figure 8**: RDe lifetime distributions at different RapA concentrations. **a)** Histogram of RDe lifetimes measured with 500nM RapA (n = 212; average = 965s ± 82.5). **b)** Histogram of RDe lifetimes measured with 100nM RapA (n = 289; average = 983s ± 61.9). **c)** Histogram of RDe lifetimes measured with 10nM RapA (n = 244; average = 1800s ± 144). **d)** Histogram of RDe lifetimes measured with 3nM RapA (n = 421; average = 3270s ± 182).


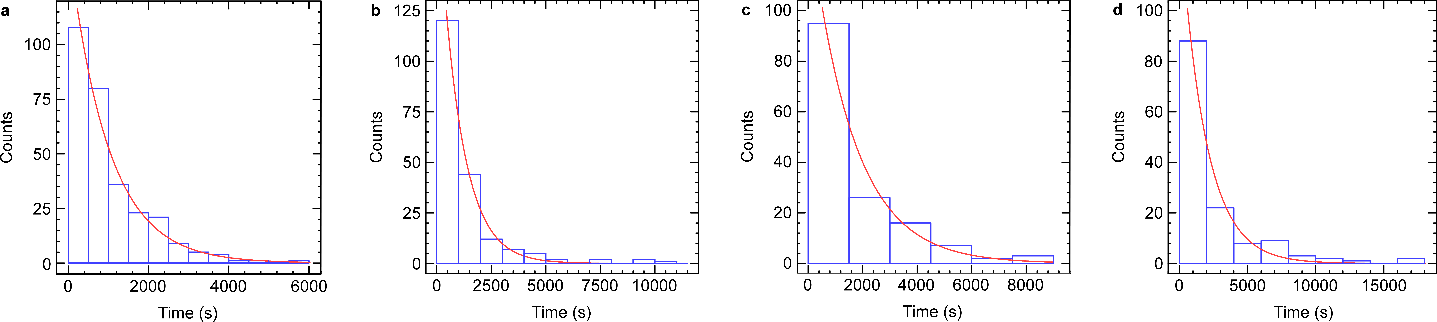


**Supplementary Figure 9**: RDe lifetime distributions at different ATP concentrations. **a)** Histogram of RDe lifetimes measured with 1mM ATP (n = 289; average = 983s ± 61.9). **b)** Histogram of RDe lifetimes measured with 400µM ATP (n = 195; average = 1080s ± 97.1). **c)** Histogram of RDe lifetimes measured with 200µM ATP (n = 149; average = 1600s ± 163). **d)** Histogram of RDe lifetimes measured with 125µM ATP (n = 135; average = 1870s ± 212).


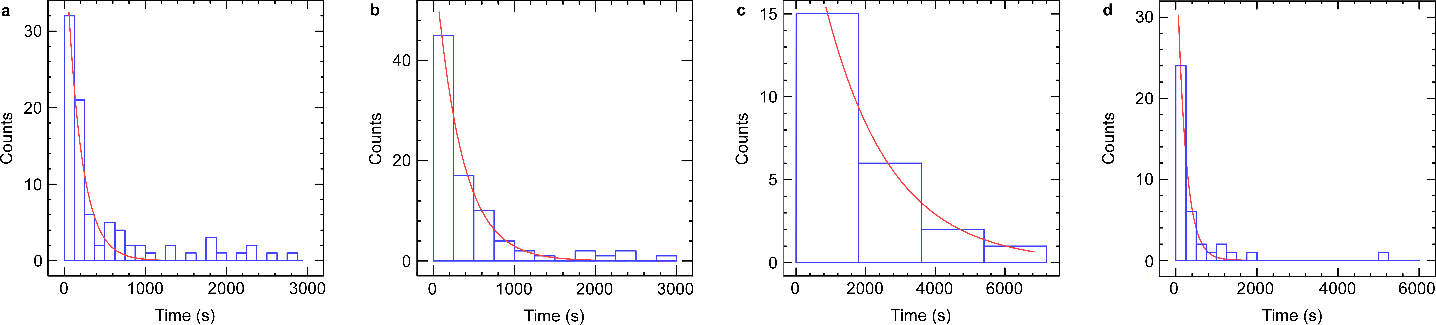


**Supplementary Figure 10**: RDe lifetime distributions at different stalling distances. **a)** Histogram of RDe lifetimes with RNAP stalling at +20 (n = 87; average = 181s ± 27.5). **b)** Histogram of RDe lifetimes with RNAP stalling at +36 (n = 85; average = 328s ± 43.4). **c)** Histogram of RDe lifetimes with RNAP stalling at +83 (n = 24; average = 1890s ± 505). **d)** Histogram of RDe lifetimes measured at 2.0pN with RNAP stalling at +20 in the presence of 100U/µl RNAse A (n = 38; average = 215s ± 45.5).
